# An electronic diary on a palm device for headache monitoring: a preliminary experience

**DOI:** 10.1007/s10194-012-0473-2

**Published:** 2012-07-28

**Authors:** Marta Allena, Maria Giovanna Cuzzoni, Cristina Tassorelli, Giuseppe Nappi, Fabio Antonaci

**Affiliations:** 1 Headache Science Centre, C. Mondino National Institute of Neurology Foundation IRCCS, Pavia, Italy; 2 University Consortium for Adaptive Disorders and Head pain (UCADH), Varese, Italy; 3 University Consortium for Adaptive Disorders and Head pain (UCADH), Pavia, Italy

**Keywords:** Medication overuse headache, Electronic diary, Headache, Feasibility, Compliance

## Abstract

Patients suffering from headache are usually asked to use charts to allow monitoring of their disease. These diaries, providing they are regularly filled in, become crucial in the diagnosis and management of headache disorders because they provide further information on attack frequency and temporal pattern, drug intake, trigger factors, and short-/long-term responses to treatment. Electronic tools could facilitate diary monitoring and thus the management of headaches. Medication overuse headache (MOH) is a chronic and disabling condition that can be treated by withdrawing the overused drug(s) and adopting specific approaches that focus on the development of a close doctor–patient relationship in the post-withdrawal phase. Although the headache diary is, in this context, an essential tool for the constant, reliable monitoring of these patients to prevent relapses, very little is known about the applicability of electronic diaries in MOH patients. The purpose of this study was to evaluate the acceptability of and patient compliance with an electronic headache diary (palm device) as compared with a traditional diary chart in a group of headache inpatients with MOH. A palm diary device, developed in accordance with the ICHD-II criteria, was given to 85 MOH inpatients during the detoxification phase. On the first day of hospitalization, the patients were instructed in the use of the diary and were then required to fill it in daily for the following 7 days. Data on the patients’ opinions on the electronic diary and the instructions given, its screen and layout, as well as its convenience and ease of use, in comparison with the traditional paper version, were collected using a numerical rating scale. A total of 504 days with headache were recorded in both the electronic and the traditional headache diaries simultaneously. The level of patient compliance was good. The patients appreciated the electronic headache diary, deeming it easy to understand and to use (fill in); most of the patients rated the palm device handier than the traditional paper version.

## Background

Patients suffering from headache are usually asked to use charts to allow monitoring of their disease. These diaries, which must be filled in regularly, have a twofold aim: to facilitate both the diagnosis and the management of headache disorders by providing prospective and, therefore, more reliable information concerning the frequency and temporal pattern of attacks, intake of drugs, trigger factors, and responses to treatment [1]. Until now, paper diaries and calendars for recording headache attacks, usually in the form of booklets, have been the most widely used instruments in clinical practice for the management of headache patients [2]. These diaries, which are filled in at home by the patients and returned at follow-up visits, allow prospective recording of the characteristics of every attack and therefore more accurate descriptions of the disease; they also make it possible to confirm and/or distinguish between different types of headache in subjects with coexisting forms.

In a recent study by our group, conducted in collaboration with the Danish Headache Centre, we evaluated the applicability and usefulness of a basic paper diary in assisting the diagnosis of migraine and tension-type headache [3]. The basic headache diary, which had been given to the patients before their first consultation at the Headache Centre, was found, at the clinical interview, to provide additional information useful for making the diagnosis and establishing the pain pattern. It was well accepted and adequately filled in by the patients.

Despite its many advantages, the paper diary has some important limitations, for example, patients, instead of filling it in regularly on a daily basis, may complete multiple entries at the same time, thereby introducing systematic bias due to retrospective recall that could interfere with the validity of the data collected. Furthermore, paper diaries can be lost, are more likely to be forgotten and cannot be visualized remotely by the doctor.

The Internet and new technologies have evolved remarkably in recent years and they are increasingly used by patients as a means of obtaining health-related information and of contacting members of the medical profession.

It can be hypothesised that electronic tools could facilitate the diary monitoring of headache and thus the management of headache conditions. Over the past decade, electronic diaries have been used to assess migraine symptoms in headache patients, to record non-headache symptoms occurring before, during and after migraine attacks [4], to evaluate the occurrence of and relationship between headaches and premenstrual syndrome symptoms [5], and also to monitor the efficacy of migraine treatments in clinical trials [6, 7]. The use of electronic diary devices may also improve patient compliance as suggested by Stone et al. [8], who compared an electronic diary with a paper version.

In 2007, Sorbi and colleagues [9] published the results of a pilot study conducted to test the feasibility and acceptability of a new method for mobile Web-based monitoring and coaching in a small group of chronic migraine patients. They evaluated the use of an online digital assistance (ODA) tool intended as a support for home-based training of cognitive behavioural treatment in chronic migraine; ODA combines mobile coaching with diary monitoring.

More recently, other authors explored, in migraine patients, the feasibility and acceptability of an internet-based headache diary, compared with their standard paper diary [10]. This study showed that the internet-based headache diary was not only a feasible and acceptable data collection tool, but also an accessible option for different populations.

Some years ago, at the Headache Centre of the C. Mondino National Institute of Neurology in Pavia, we developed a simple electronic web-diary running on Excel algorithms. Designed for use in headache patients, this instrument was tested on a small sample of subjects with episodic headache and gave satisfactory results [11].

In patients with a chronic pattern of headache, the diary becomes an essential tool for the monitoring and, in such cases, preventing relapses. Particularly, Medication Overuse Headache (MOH), a common chronic and disabling condition, is considered one of the most frequent forms of headache seeking help at the Headache Centres and it represents a challenge for clinicians [12]. Its treatment leads to improvement in up to 75 % of patients, but the relapse rate may exceed 40 %. Often patients suffering from MOH do not have regular contact with health-care providers or stop seeing physician for their headache because of the unsuccessful outcome of the disease (i.e. relapsing in overuse of acute medications or the persistence of a chronic pain) [13].

Then, MOH represents a perfect example of a disorder that can benefit from an electronic headache diary, by improving and integrating the traditional paper, for a more adequate and continuous monitoring of disease evolution and its successful management.

However, very little is known about the applicability of electronic diaries in MOH patients.

Recently, we developed a specific protocol for the management of MOH and showed that closer monitoring of these patients, achieved through more frequent visits (once every 2–4 months) and easier access to physicians during the post-detoxification phase, might indeed favour a better outcome with a reduced rate of relapses [14]. To further improve and build on this successful care approach, we decided to create and test an electronic diary running on a palm device (digital headache diary, DHD) to encourage the contact between headache sufferers and health care.

In the present study, we report the results obtained from testing the DHD in a group of MOH inpatients to evaluate the acceptability of and patient compliance with this instrument compared with our traditional diary chart. Further step will be to develop an electronic and interactive tool for the management of patients with this disabling disorder.

## Materials and methods

MOH patients were recruited for the study during the detoxification phase, according to the protocol described in detail elsewhere [12] and applying the ICHD-II criteria [15]. During the hospitalization period (usually 5–7 days), patients were given the palm device with the diary (DHD) along with the conventional paper diary usually used in our hospital.

### Device

**Fig. 1:**
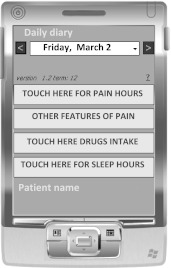
Example of a DHD developed from the paper one

**Fig. 2:**
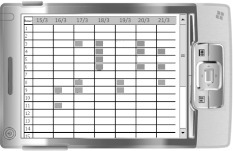
Example of a HDD weekly report

The palm device (ATC Service—Pavia, Italy) is a personal digital assistant (PDA) with 32 MB RAM, a colour screen and Windows CE version 3.0 operating system (Figs. 1, 2).

### Electronic headache diary

The DHD was developed by ATC according to the requirements of and under the supervision of one of the authors (FA); it was derived (as a simplified version) from the paper diary currently in use at our hospital and based on the ICHD-II diagnostic criteria [12]. A set of simple but detailed instructions was also created. The e-diary was structured as a calendar in which the dates were already printed. Diary recording periods consisted of 30 consecutive days, and the instrument offered the possibility of visualizing daily/weekly/monthly reports (Fig. 2 shows a weekly report) uploadable onto a personal computer via a synchronization cable.

The DHD is divided into three sections covering features of headache, medication use, and trigger factors. In the first section, the patient is required to note, for each headache day, sleep time (hours), presence of aura symptoms, time of pain onset and features of pain (intensity, side, type, presence of associated symptoms). The second and third sections of the diary investigate intake of painkillers (type of drug/s, time of intake and total number of doses taken during the 24 h) and try to identify possible trigger factors of the headache attack.

After receiving on admission appropriate instruction on the use of the diaries, the patients filled them in on a daily basis; in this way, we were able to collect detailed information on headache attacks and drug use.

Upon discharge, all the patients were asked to evaluate and compare the two versions of the diary (paper vs. electronic), rating a series of items using a 10-point numerical rating scale (0, not at all; 10, very). In short, they were asked to decide whether the instruments were easy to understand, easy to use and handy, and also to rate the visual impact of the layouts. The form also included an open space for comments, problems, suggestions and the patient’s overall positive or negative evaluation of the system.

The measured variables were expressed as mean values ± SD. For comparison of the ratings assigned to the paper diary versus the DHD, data were analysed using the Student’s paired *t* test.

## Results

We analysed data from a total of 85 enrolled patients, comprising 68 females and 17 males, with a mean age of 39.7 ± 10.2 years and a relatively high level of education (mean years of education 12.2 ± 3.6) (Table 1). A total of 504 days with headache showing comparable features (intensity, type of pain, associated symptoms and drug intake) were recorded in both diaries simultaneously, during a recording period of 7–10 days for each patient.

**Table 1:** Socio-demographic variables and headache parameters of headache patients included in the study

	Patients (*n* = 85)
Sex (F/M)	68/17
Age (years)	39.73 ± 10.2
Education (secondary school or higher)	69.4 %
MOH duration (months)	42.2 ± 37.6
First detoxification	64.7 %

The mean duration of MOH was 42.2 ± 37.6 months, while the mean scores on the MIDAS and HIT-6 scales (two simple questionnaires designed to measure headache-related disability) were very high (83.5 ± 60.3 and 66.5 ± 6.7, respectively), confirming the high level of disability associated with this chronic disorder.

Patient compliance with the DHD was very good: the electronic handheld diaries were completely filled in by 98 % of the patients during their hospital stay. Age, educational level and baseline headache disability were not found to influence diary completion. The instructions were rated adequate and clear by 97 % of subjects. As regards the subjective evaluations, the patients gave the readability of the screen and the design of the layout positive ratings. The DHD was rated easier to understand (*p* < 0.01) and easier to use (*p* < 0.0002) than the paper diary. The electronic diary scored significantly higher in all the items, as compared with the paper version (*p* < 0.01) (Table 2).

**Table 2:** Comparison of patients’ subjective evaluations (ratings) of headache diaries

Item	Paper diary (m ± SD)	Palm diary (m ± SD)	P*
Easy to understand	8.3	8.7	<0.01
Easy to use	7.9	8.9	<0.01
Handy	8.2	8.9	<0.01
Visual impact of the layout	7.8	8.5	<0.01
Overall preference	7.4	8.3	<0.01

* Student’s paired *t* test

Furthermore, all the patients but one preferred the DHD to the paper version. The one patient who preferred the paper diary, deeming it more convenient and easier to use, was completely naïve to electronic tools.

It is noteworthy that a trend towards significance (*p* = 0.05) was observed when analysing patients who had previously failed on detoxification programmes; furthermore, this subgroup of patients showed a lower DHD completion rate.

To improve the system, some participants suggested including more options for reporting headache features, for example the addition of a drawing for indicating pain location or the use of a free-text section to allow better descriptions of headache intensity and type, as well as associated symptoms. Some patients also recommended a more extensive drop-down list of acute drugs taken where they might directly select the drug used.

## Conclusions

In this study, we assessed the acceptability of a DHD, compared with a traditional paper diary, in a population of MOH inpatients recruited while in hospital undergoing detoxification.

The patients readily accepted the electronic headache diary, deeming it easy to understand and to use. Diary compilation was found to be complete (no information missing) in 98 % of cases.

On the evaluation scale, patients expressed their very positive impression of the DHD and of its convenience, and reported no difficulties in understanding or inputting the required information. Most of the patients considered the palm device handier than the traditional paper version, and they also asked whether the software could be installed on their smartphone or on their personal computer at home.

The electronic diary was also found to be convenient by the physicians involved in this study, who believe that it could be an extremely practical and helpful instrument for clinical headache monitoring. Indeed, the generation of the final report allows storage of data useful for clinical practice (follow ups) as well as for research purposes.

A possible limitation of our study is that the electronic diary was given to a selected subgroup of subjects with a higher mean level of education who received, from professionals, face-to-face instruction in its use.

Paper-and-pencil diaries are commonly used in headache care and clinical research to assess patient pain experiences. They are easy to use and usually well received by headache patients and physicians. Our electronic headache diary, programmed into a pocket computer, represents a simple and promising method for clinical headache monitoring, not least due to its other possible multifaceted applications, which include the creation of an easily downloadable application for smartphones, suitable for adoption on a large scale, and the possibility of building a more complex logic, transforming the diary, through the integration of more complex functionalities, into a structured and guided system of communication between patient and physician.

New and handy strategies, such as our HDD, associated to the education of the patients on their disabling disease, need to be devised to improve long-term outcome of MOH. Further studies are currently under way to confirm the multiple potentialities of such electronic tools in the management of severe headaches.

## References

[1] Nappi G, Jensen R, Nappi RE, Sances G, Torelli P, Olesen J (2006). Diaries and calendars for migraine. A review. Cephalalgia.

[2] Torelli P, Jensen R (2010). Headache diaries and calendars. Handb Clin Neurol.

[3] Tassorelli C, Sances G, Allena M, Ghiotto N, Bendtsen L, Olesen J, Nappi G, Jensen R (2008). The usefulness and applicability of a basic headache diary before first consultation: results of a pilot study conducted in two centres. Cephalalgia.

[4] Giffin NJ, Ruggiero L, Lipton RB (2003). Premonitory symptoms in migraine. Neurology.

[5] Goldberg J, Wolf A, Silberstein S, Gebeline-Myers C, Hopkins M, Einhorn K, Tolosa JE (2007). Evaluation of an electronic diary as a diagnostic tool to study headache and premenstrual symptoms in migraineurs. Headache.

[6] van Gerven JM, Schoemaker RC, Jacobs LD, Reints A, Ouwersloot-van der Meij MJ, Hoedemaker HG, Cohen AF (1996). Self-medication of a single headache episode with ketoprofen, ibuprofen or placebo, home-monitored with an electronic patient diary. Br J Clin Pharmacol.

[7] Mathew NT, Frishberg BM, Gawel M, Dimitrova R, Gibson J, Turkel C, BOTOX CDH Study Group (2005). Botulinum toxin type A (BOTOX) for the prophylactic treatment of chronic daily headache: a randomized, double-blind, placebo-controlled trial. Headache.

[8] Stone AA, Shiffman S, Schwartz JE, Broderick JE, Hufford MR (2003). Patient compliance with paper and electronic diaries. Controlled Clin Trials.

[9] Sorbi MJ, Mak SB, Houtveen JH, Kleiboer AM, van Doornen LJ (2007). Mobile web-based monitoring and coaching: feasibility in chronic migraine. J Med Internet Res.

[10] Moloney MF, Aycock DM, Cotsonis GA, Myerburg S, Farino C, Lentz M (2009). An internet-based migraine headache diary: issues in internet-based research. Headache.

[11] Sances G, Tassorelli C, Ghiotto N, Loi M, Guaschino E, Pagani M, Corti L, Nappi G (2007) The development of an electronic web-diary for the monitoring of primary headaches. In: Jensen R, Diener HC, Olesen J (eds) Headache clinics: organization, patients and treatment. Frontiers in headache research. Oxford University Press, Oxford, pp 157–162

[12] Steiner TJ, Birbeck GL, Jensen R, Katsarava Z, Martelletti P, Stovner LJ (2011) The Global Campaign, World Health Organization and Lifting The Burden: collaboration in action. J Headache Pain 12(3):273–4 10.1007/s10194-011-0342-4 PMC3094667 21512775

[13] Jonsson P, Linde M, Hensing G, Hedenrud T (2012) Sociodemographic differences in medication use, health-care contacts and sickness absence among individuals with medication-overuse headache. J Headache Pain 13(4):281–90 10.1007/s10194-012-0432-y PMC3356474 22427000

[14] Ghiotto N, Sances G, Galli F, Tassorelli C, Guaschino E, Sandrini G, Nappi G (2009). Medication-overuse headache and applicability of the ICHD-II diagnostic criteria: 1-year follow-up study (CARE I protocol). Cephalalgia.

[15] Headache Classification Subcommittee of the International Headache Society (2004). The International Classification of Headache Disorders, 2nd edition. Cephalalgia.

